# Exploring botulinum toxin’s impact on masseter hypertrophy: a randomized, triple-blinded clinical trial

**DOI:** 10.1038/s41598-024-65395-5

**Published:** 2024-06-24

**Authors:** Bryanne Brissian de Souza Nobre, Luciana Rezende, Mariana Barbosa Câmara-Souza, Alfonso Sanchez-Ayala, Rodrigo Blass, Ana Claudia Carbone, Ana Cristina Manso, Malin Ernberg, Nikolaos Christidis, Giancarlo De la Torre Canales

**Affiliations:** 1 Department of Dentistry, Ingá University Center, Uningá, Paraná Brazil; 2 Department of Dentistry, University of Ponta Grossa, Ponta Grossa, Paraná Brazil; 3 Private Practice, Sao Paulo, Brazil; 4 Egas Moniz Center for Interdisciplinary Research (CiiEM), Egas Moniz School of Health & Science, Caparica, Almada, Portugal; 5 Division of Oral Diagnostics and Rehabilitation, Department of Dental Medicine, Karolinska Institutet, and the Scandinavian Center for Orofacial Neurosciences (SCON), 14104 Huddinge, Sweden

**Keywords:** Botulinum toxin type A, Masseter hypertrophy, Muscle thickness, Masticatory performance, Outcomes research, Skeletal muscle

## Abstract

The present study aimed to assess the effectiveness and functional adverse effects of a single and multiple injections of botulinum toxin A (BoNT-A) for masseter hypertrophy (MH). Twenty-six women complaining about lower third facial enlargement due to MH, received 75 U of BoNT-A (abobotulinum toxin) in each masseter muscles. After 3 months, patients were randomly assigned to receive a second treatment session of Saline Solution: (G1; n = 11) or BoNT-A: (G2; n = 12). Muscle thickness (ultrasound), electrical activity (electromyography; EMG), masticatory performance, and subjective perception of MH were evaluated. Follow-up was performed at 1, 3 and 6 months. Muscle thickness, EMG activity, and masticatory performance were analyzed using ANOVA two-way and Sidak test as post-hoc. Masticatory performance was analyzed by the Friedman’s test and Mann–Whitney test. Regarding inter-groups comparisons, there was a significant decrease in the left masseter muscle thickness in the G2 group at the 6 month follow-up (p < 0.02). For EMG, significant differences were evident at the 6 month assessment, with higher masseter activity for G1 (p < 0.05). For masticatory performance, no significant differences were observed throughout the study (p > 0.05) and a higher improvement in subjective perception of MH was observed in the 1 month follow-up for G2 (p < 0.05). In conclusion, BoNT-A is effective for MH, however multiple injections cause functional adverse effects in masseter muscle.

## Introduction

The perception of aesthetics is influenced by cultural preferences. However, certain principles of beauty appear to have universal applicability^1^. Universal attributes of beauty and attractiveness include sexual dimorphism, youthfulness, symmetry, and facial proportions^1,2^. For women, a triangular face shape is often considered attractive, characterized by a narrower lower third of the face compared to men. The lower third of the face is determined by the jawbone and soft tissues, such as the skin, subcutaneous tissue, and the masseter muscle. Therefore, a wider lower third of the face, commonly referred to as a square face, is mainly influenced by the mandibular angle and masseter muscle hypertrophy (MH)^3^.

MH is characterized by the asymptomatic enlargement of the masseter muscles, which can occur unilaterally or bilaterally. It is more commonly observed in individuals aged 20–40, with no specific gender predilection^4^. While its etiology remains unknown, several factors, including emotional stress, chronic bruxism, microtraumas, prognathism, hyperfunction, and the use of neuroleptics, have been linked to this condition^5,6^. Common treatments for MH include surgical intervention, which involves the partial removal of the masseter muscle and bone adjustments in the region of the jaw angle, as well as conservative minimally invasive treatments such as injection of botulinum toxin A (BoNT-A). However, both types of treatment pose risks, such as injury to the facial nerve and fracture of jaw structures with surgery, and a reduction in muscle function with BoNT-A treatment^7^.

BoNT-A is a neurotoxin produced by the bacteria *Clostridium botulinum*, capable of inducing paralysis by blocking the release of acetylcholine in neuromuscular junctions^8^. This mechanism of action results in muscle thinning (atrophy), leading to the conclusion from clinical trials that BoNT-A is an effective treatment for MH^9–11^. However, systematic reviews regarding the effectiveness of this treatment for MH have highlighted the insufficient high-quality evidence needed to definitively establish BoNT-A’s effectiveness for this condition^7,12, 13^. Additionally, experimental studies have observed that the induction of localized masticatory atrophy by BoNT-A injection alters the muscle’s histological composition, resulting in increased mRNA levels of molecular markers associated with atrophy (e.g. Atrogin-1/Mafbx and MuRF-1), neurogenic atrophy, and a reduction in muscle’s fibers diameter and muscle mass^14–17^. Furthermore, the replacement of type IIa muscle fibers (anti-fatigue) for type IIb muscle fibers (non-anti-fatigue) and the replacement of contractile tissue with fat have also been observed in animal studies^14–16^. In addition, animal literature has found that these muscular alterations could worsen if we consider repeated injections of the treatment in masticatory muscles and as consequence lead to changes in mandibular bone which have been also demonstrated in clinical trials^18–20^.

Clinical studies have confirmed the occurrence of adverse effects on muscle tissue following the administration of BoNT-A in patients with MH. These temporary adverse effects include muscle weakness^21^, a reduction in maximum bite force of up to 20% and a decrease in masticatory performance^22^. However, a recent study that evaluated the adverse effects of BoNT-A injection into the masseter muscle of patients with myofascial TMD pain, observed that a single dose of 30U of onabotulinumtoxinA caused a reversible decrease in masticatory performance (using multiple sieve method) but not a decrease in masseter muscle thickness^23^, in contrast to repeated injections that led to a severe diminution of masseter muscle thickness that still lasted four years after the last BoNT-A injection^24^. Collectively, these results indicate that muscle atrophy increases with the number of applications, irrespective of the administered doses. It is important to note that the latter study did not evaluate any functional adverse effects, underscoring the need for a comprehensive longitudinal analysis of BoNT-A’s effectiveness and adverse effects ratio for MH. Furthermore, an unresolved question remains: is it worthwhile to affect such an important muscle as the masseter muscle solely for aesthetic purposes?

Therefore, this study aimed to longitudinally assess the effectiveness and functional adverse effects of a single and multiple injections of BoNT-A as treatment for MH.

## Methods

This research received approval from the Research Ethics Committee of Uningá University (CAAE: 63135022.3.0000.5220) and the Brazilian Registry of Clinical Trials (RBR-7tdjcn5–24/01/2024). All participants were thoroughly informed about the research objectives and provided written consent to take part in the clinical trial. The study, which was longitudinal, triple-blind, and placebo-controlled, took place at the dental clinic of the Associação Brasileira de Odontologia in Goiás-Goiânia (ABO-Goias) between March 2 and October 17, 2023, and adhered to the Helsinki Declaration. The reporting of data followed the CONSORT checklist.

### Participants

The sample comprised Brazilian women aged 25–50 years, experiencing lower third facial enlargement due to MH at severity levels II to V on the 10-point photonumeric masseter prominence scale—Merz^25^. Exclusion criteria encompassed patients who had previously received BoNT-A injections for aesthetic or therapeutic purposes, facial fillers in any facial region, any facial surgery, missing teeth, autoimmune and neuromuscular diseases, pregnancy or breastfeeding, and any type of vaccine within 3 months prior to the study commencement. The sample size calculation was based on a previous study^23^. That study reported an average change of masseter muscle thickness of 25% (± 8%) after BoNT-A injections. Power calculation showed that with this data, nine patients per group would demonstrate a β = 0.9 and α = 0.01. However, considering the longitudinal nature of the study, which could lead to possible dropouts of volunteers, an additional four participants were included per group. The total sample size included in the presented study was of 26 patients divided in two groups of 13 patients each.

### Study protocol

Participants underwent five assessments during the study. During the initial visit, they were screened based on the study’s inclusion and exclusion criteria. Those included were informed about the study protocol, treatments, and assessment methods. They were informed about receiving two injection sessions with a 3 month interval: the first session involved BoNT-A, while the second session included either BoNT-A or a saline solution. Importantly, neither the researcher administering the injections, the researcher assessing the outcome, nor the volunteers themselves were aware of the administrated treatment in the second injection session. At the second visit, baseline variables assessments were conducted, and BoNT-A injections were administered. A follow-up examination (Visit 3) took place 1 month after the first injection. Three months after the first injection (Visit 4) patients were randomly assigned to receive saline solution (G1; n = 11) or BoNT-A (G2; n = 12). Then, 6 months after the first injection session a final follow-up took place (Visit 5).

### Randomization and blinding

Randomization was performed using an internet-based computer program (http://www.randomization.com/) in blocks of four patients by a technician not involved in any other procedure of the study. For each patient, the technician placed a note with the randomized treatment (BoNT-A or saline) in a sealed opaque envelope. The randomization list was kept by the technician in a locked drawer/cabin during data sampling and was not revealed to the researchers until after data sampling was finished. Thus, the block size and randomization were unknown to the researcher injecting the substances (L.R.) and the researcher examining the patients (B.B.S.N.), as well as to the patients. The opening of the envelopes was done just before injections by a third researcher (A.C.C.) only involved in the preparation of the syringes. The syringes were marked with patient code and left in the examination room before the researcher giving the injections (L.R.) and patient entered. BoNT-A and SS are colorless solutions with identical appearance.

### Interventions

The BoNT-A injection protocol involved reconstituting BoNT-A vials (Dysport^®^ (abobotulinumtoxinA) 500 U Ipsen, Wrexham, United Kingdom) with 2 mL of 0.9% isotonic sterile saline solution, stored at room temperature, giving a dose of 25 U/0.1 mL. This dose of abobotulinumtoxinA is similar to dose of 10 U/0.1 mL of onabotulinumtoxinA^26–28^. The total doses administered in each masseter muscle was 75 U divided into three injection points (25 U each) bilaterally in the masseter muscles, totaling 150 U for each patient. Additionally, a total of 0.6 mL of saline solution (0.3 mL/side) served as the control. Injections were conducted in a secure area delineated by a line from the tragus to the corner of the mouth, a second line located 0.5 cm above the lower edge of the mandible, and within two vertical lines marking the anterior and posterior belly of the masseter muscle after a functional test (teeth clenching). Both the BoNT-A and saline solution are colorless solutions with distinguishable appearances and were prepared by a trained researcher. The injections were administered by an experienced specialist in injectable facial aesthetic procedures, not involved in any other study procedures. Injections were done using 1 mL syringes with 13 × 0.26 mm (26G) needles.

### Outcomes

Patients’ outcomes were assessed at four time points (baseline, 1, 3, 6 months) over the 6 month study period. Both subjective assessments and objective measurements were conducted at each evaluation period by a researcher (B.B.S.N.) not involved in any other study procedures. For the subjective assessment the ten-point photonumeric masseter prominence scale—Merz^25^ was used, while muscle thickness measured with ultrasound (US) and electromyographic (EMG) recordings constituted the objective evaluations. Muscle thickness was considered as the primary outcome for this study.

#### Ultrasound

The thickness of both masseter muscles during maximum voluntary contraction (MVC) was measured using real-time US (A6-ultrasound^®^ machine-transducer model L745 with a standard linear array (40 mm) p 11.0–5.0 MHz, SonoScape, China) by a single calibrated operator. Intra-observer agreement was assessed with Cohen’s Kappa coefficient for two measurements on the same cases (n = 3), using US imaging (Kappa = 0.86). Patients were positioned in a supine position at an ergonomically favorable height for the examination. Muscle thickness measurements were taken with the recording location determined by palpation, following the same anatomical references used for treatment injections (mid-level between the zygomatic arch and the gonial angle, close to the occlusal plane). Measurements were directly performed on the instrument’s screen (with an accuracy of 0.01 mm), corresponding to the most voluminous part of the muscle image. The final value was obtained by averaging the three measurements in the same position^29^.

#### Electromyography

To record the EMG signal, a four-channel EMG system (Miotool NG USB^®^, Porto Alegre, RS, Brazil, frequency range: 10–700 Hz; sampling rate 3000/s; resolution: 2.44 V/bit) was utilized by a single-calibrated operator. Bipolar surface electrodes (Ag–AgCl disks, Covidien llc, Quebec, Canada) were fixed to the masseter muscles (using the same anatomical references as for treatment injections) after cleansing the skin with cotton and 70% alcohol to minimize impedance at the electrode sites. The reference electrode was positioned on the manubrium of the sternum. Masseter muscle electrical activity was recorded during MVC. Patients were seated in a chair with their head and shoulders held straight in a relaxed position, and the Frankfurt plane parallel to the floor. Patients were then instructed to clench their teeth with maximum force and maintain this for 5 s. This process was repeated three times with a 2 min rest period between collections to prevent fatigue^30^. The complete EMG signal was captured at a frequency of 1000 Hz, followed by band-pass filtering for 20–500 Hz to obtain the root-mean-square (RMS) value pertaining to 5 s of MVC of the masseter muscles. MiotecSuite software 1.0 (Miotec Equipamentos Biomédicos, Porto Alegre, Brazil) was employed for data analysis, and the mean of the three MVC recordings was used for statistical analyses.

#### Masticatory performance

To assess masticatory performance, participants were instructed by a trained researcher to chew a two-colored chewing gum (Vivident Fruitswing Karpuz, Turkey) for 20 chewing cycles. Subsequently, the chewed gum was placed in a transparent plastic bag and flattened to a thickness of 1 mm. Both sides of the flattened gum were scanned at 300 dpi, and the resulting images were imported into the ViewGum^©^ program for electronic colorimetric analysis. Inadequate mixing of the gum colors led to a greater variance of hue (VOH), indicating poorer masticatory performance^31^.

#### Masseter prominence

The severity of masseter prominence was evaluated using a 10-point photonumeric scale—Merz specifically developed for females, ranging from I (none) to V (much), and also indicating the position of the maximum masseter prominence (low or high)^29^.

### Statistical analysis

Data were assessed for distribution normality by using the Shapiro–Wilk test. As data for masseter thickness did not present normal distribution, data were submitted to Log10(x + 1) transformation, and parametric tests were used. US, EMG, and MP were analyzed by using ANOVA two-way with repeated measures and Sidak test as post-hoc. Also, data from the photonumeric scales were not normally distributed and therefore analyzed with the Friedman’s test (intragroup comparisons) and Mann–Whitney test (intergroup comparisons). The SPSS software (IBM Corp. Released 2021. IBM SPSS Statistics for Windows, Version 28.0. Armonk, NY: IBM Corp) was used to run all test, and the significance level was set at p < 0.05.

## Results

**Figure 1:**
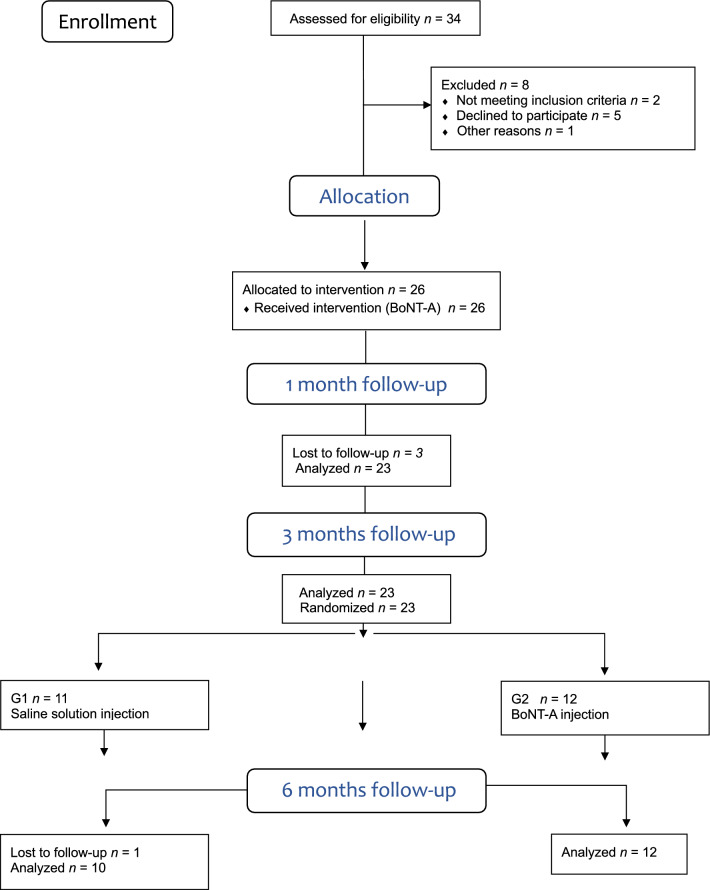
CONSORT flow diagram.

In total, 34 patients underwent screening; eight (23.5%) did not meet the inclusion criteria, leading to the enrollment of 26 patients (32.5 ± 8.04) in this trial (Fig. 1). However, two patients from G1 and one from G2 withdrew from the study before the 1 month follow-up and one more patient before the final follow-up, resulting in a total of 22 patients completing the study (G1: n = 10 and G2: n = 12). No significant differences in age were observed between the groups (p > 0.05) (Fig. 1).

### Muscle thickness

#### Right masseter

**Figure 2:**
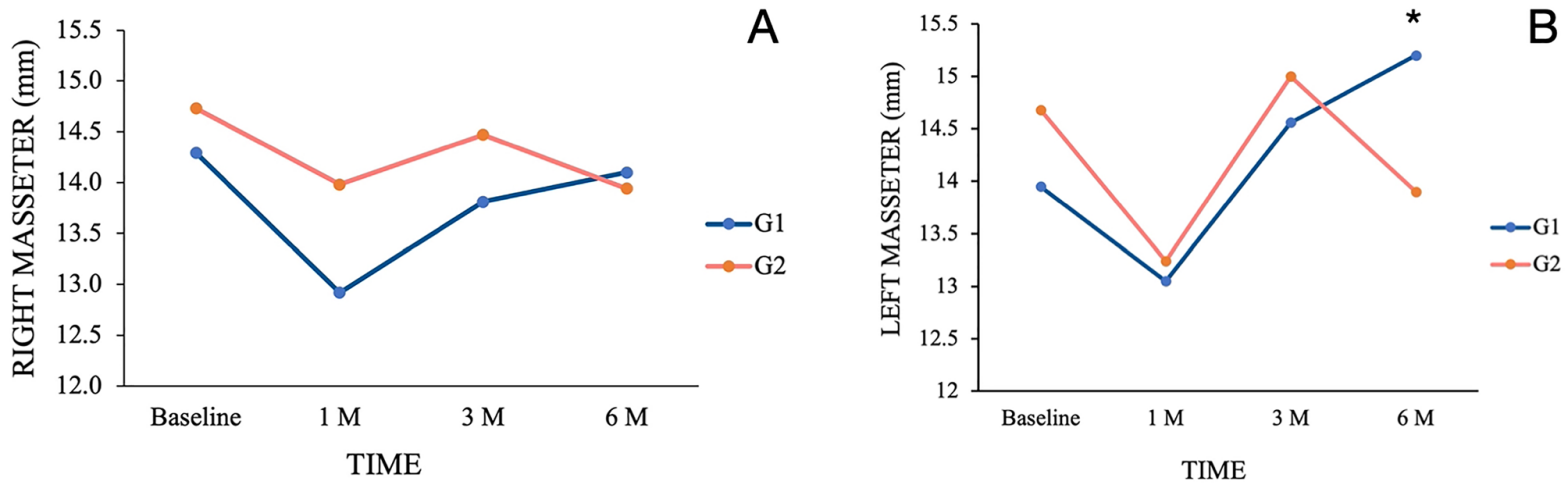
Changes in muscle thickness (mm) during maximum voluntary contraction (MVC) for each group in different time points. Intergroups differences: *p < 0.02 (left masseter).

Intra-group comparisons showed a significant decrease in masseter muscle thickness during MVC when comparing baseline assessment with the 1 month assessment within G1 and G2 (p < 0.05) (G1: Baseline: 14.29 ± 1.73 mm; 1 M: 12.92 ± 2.61 mm; 3 M: 13.81 ± 1.74 mm; 6 M: 14.10 ± 1.85 mm / G2: Baseline: 14.73 ± 2.14 mm; 1 M: 13.68 ± 2.31 mm; 3 M: 14.47 ± 2.10 mm; 6 M: 13.94 ± 1.61 mm). Similarly, there were no significant differences found in the inter-group comparisons throughout the entire study (p > 0.05) (Fig. 2A).

#### Left masseter

A significant decrease was found in both groups when comparing the baseline assessment and the 1 month follow-up (p < 0.05) in the intra-group comparisons (G1:Baseline: 13.95 ± 1.67 mm; 1 M: 13.05 ± 2.30 mm; 3 M: 14.56 ± 2.19 mm; 6 M: 15.20 ± 1.45 mm / G2: Baseline: 14.68 ± 2.23 mm; 1 M: 13.24 ± 2.81 mm; 3 M: 15.00 ± 1.14 mm; 6 M: 13.90 ± 2.45 mm). However, a significant increase in masseter muscle thickness was observed only in G1 when comparing the 1 month and 6 month follow-ups (p < 0.01). Inter-group comparisons revealed a significant lesser masseter muscle thickness in G2 than in G1 at the 6 month follow-up (p < 0.02) (Fig. 2B).

### Electromyographic activity

#### Right masseter

**Figure 3:**
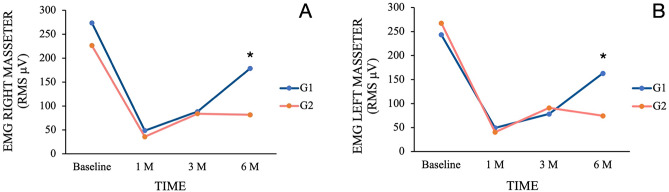
Changes in root mean square scores (RMS μV) in maximum voluntary contraction (MVC) for each group in different time points. Intergroups differences: *p < 0.005 (left masseter); *p < 0.04 (right masseter). *EMG* electromyography.

Intra-group comparisons (G1: Baseline: 273.72 ± 174.64 μV; 1 M: 48.63 ± 20.29 μV; 3 M: 88.39 ± 48.89 μV; 6 M: 178.83 ± 93.15 μV / G2: Baseline: 226.40 ± 103.09 μV; 1 M: 35.89 ± 15.69 μV; 3 M: 83.99 ± 42.39 μV; 6 M: 81.77 ± 48.69 μV) revealed significant reductions in muscle activity of the right masseter after 1 month (p < 0.02) for G1, and across all follow-up periods for G2 (p < 0.001) when compared with baseline. Conversely, a significant increase in muscle activity was noted at the 6 month follow-up when compared with the 1 month (p < 0.004) and 3 month (p < 0.006) assessments in G1. As for G2, this increase was observed solely in the comparison between the 1 month and 6 month follow-ups (p < 0.044). In inter-group comparisons, significant differences were observed only in the final follow-up, with higher masseter activity found in G1 (p < 0.005) (Fig. 3A).

#### Left masseter

In the intra-group comparisons (G1:Baseline: 243.23 ± 145.11 μV; 1 M: 49.44 ± 51.85 μV; 3 M: 78.23 ± 58.86 μV; 6 M: 163.12 ± 118.70 μV/G2: Baseline: 267.54 ± 117.01 μV; 1 M: 40.38 ± 15.98 μV; 3 M: 91.21 ± 34.16 μV; 6 M: 74.49 ± 48.29 μV), BoNT-A led to a significant decrease in muscle activity of the left masseter after 1 month (p < 0.05) and 3 months (p < 0.13) in G1, and consistently across all follow-up periods for G2 (p < 0.002) in comparison to baseline. Conversely, a higher muscle activity was observed at the 6 month follow-up when compared with the 1 month (p < 0.003) and 3 month (p < 0.018) assessments in G1. However, despite a significant increase in muscle activity at the 3 month follow-up compared to the 1 month assessment in G2 (p < 0.0001), no significant differences were found between the 3 month and 6 month follow-ups (p > 0.554) in this group. In the inter-group comparisons, significant differences were evident solely in the 6 month assessment, with higher masseter activity found in G1 (p < 0.044) (Fig. 3B).

### Masticatory performance

BoNT-A led to a significant reduction in masticatory performance in both groups across all follow-up assessments compared to baseline (p < 0.03). However, no significant differences were observed between the groups throughout the entire study (p > 0.05) (Table 1).

**Table 1:** Presentation of mean (± sd) masticatory performance of each group at different assessment time-points.

	Baseline	1 month	3 months	6 months
G1	0.06 (± 0.02)*	0.27 (± 0.17)^#^	0.18 (± 0.12)^#^	0.34 (± 0.06)^#^
G2	0.05 (± 0.01)*	0.16 (± 0.13)^#^	0.24 (± 0.17)^#^	0.32 (± 0.13)^#^

Data is presented in arbitrary units where higher values indicate less mixing ability, thus, worse masticatory performance.

*p < 0.01 Baseline differences compared with follow-ups.

^#^p < 0.03 Differences between follow-ups.

### Masseter prominence

When stratified for the two groups in the relaxed position (distribution in relaxed position: G1: II (n = 1); III: (n = 7); IV: (n = 4) and V: (n = 0) / G2: II: (n = 2); III: (n = 7); IV: (n = 2) and V: (n = 1)), a significant improvement was observed in G1 when comparing baseline data with the 3 month (p < 0.02) and 6 month (p < 0.01) follow-ups, and in G2 between baseline and all follow-ups (p < 0.02). Concerning the contracted position, no significant differences were found in G1 throughout the study. However, a substantial improvement was evident in G2 when comparing the baseline results with all follow-up assessments (p < 0.0001). In inter-group comparisons, a higher improvement in masseter prominence was only observed in the 1 month follow-up for G2 in the relaxed (p < 0.020) and contracted assessments (p < 0.036) (Table 2).

**Table 2:** Median (min–max) values for the 10-point photonumeric masseter prominence scale between groups at different assessment periods.

Relaxed	Baseline	1 month	3 months	6 months
G1	3 (3–4)^#^	2 (2–3.7)	3 (2–3)	3 (1–3)
G2	3 (2.5–3)*	2 (1–2)^ƒ^	2 (1–2)	2 (1–3)
Contracted				
G1	3 (2.2–4)	2.5 (2–3.7)	3 (2–3)	3 (2–4)
G2	3 (3–4)*	2 (1–2.7)^ƒ^	2 (1–2.7)	2 (1.2–2)

Baseline differences compared with follow-ups: *p < 0.02 (relaxed); *p < 0.0001 (contracted).

Baseline differences compared with 3 and 6 month follow-up: ^#^p < 0.03.

Inter-groups differences: ^ƒ^p < 0.02 (relaxed); ^ƒ^p < 0.03 (contracted).

## Discussion

The main finding of this study was that repeated low doses of BoNT-A decreases muscle thickness, the ability to contract the masseter muscle, and the masticatory performance. Even though it could be good for aesthetic purposes^10,13, 24^, this could be detrimental for the function in the long run^32–34^. If BoNT-A is only used for aesthetic purposes this study indicates that only one session with low doses seems to be adequate based on the subjective scale of masseter hypertrophy^10,13^. This is also an indication to be careful when injecting BoNT-A repeatedly since this and previous studies indicates that occlusal forces, bite forces, and masticatory performance is significantly impaired^7,22^.

A single injection of BoNT-A does not seem to affect muscle thickness (assessed both by US and by a 10-point photonumeric scale) permanently since the thickness had recovered after 3 months, which is in accordance with several previous studies^9,10, 24, 33, 35–37^. However, in contrast to the previous studies, this study also recorded the muscle thickness after a booster injection 3 months after the first and for the participants in this group the muscle thickness did not return to normal after 6 months (i.e. 3 months after the second injection). Previous animal studies and some human studies indicate that this could be a result from incomplete re-innervation of the injected area^33^, fatty infiltration^15^, fibrosis^38^, and even atrophy due necrosis of muscle fibers in mice^39^ and humans^40^.

The results from this study indicates that muscle activity is decreased by a single injection of BoNT-A, and that the effect lasted for 3 months before the muscle started to recover. This finding is in line with previous reports also showing a recovery in muscle activity. These studies demonstrated that bite and occlusal forces gradually increased and returned to normal after 3 months^22,34^. In addition, it was shown in an animal study that the electric activity returned to normal after 3 months indicating that the neuromuscular junctions were functioning normal^33^. Finally, a systematic review showed that bite forces were reduced for 3–8 weeks and then gradually had recovered after 3 months^32^. However, in contrast to previous studies, this study could show that if you give a booster injection 3 months after the first injection there is no recovery of the muscle activity at the 6 month follow-up. This indicate that neuromuscular junctions do not recover to normal function, or that this recovery either takes much longer time after a repeated injection, which consequently could be considered detrimental for muscle activity^33^. This decreased muscular activity could be a result from an incomplete recovery of the innervation in the muscle since the morphological features of the masseter muscle 12 weeks after treatment with BoNT shown by Baldwin et al. (2022) resemble those of the tibialis anterior in denervated rabbits^41^.

When it comes to masticatory performance this study indicates that it is impaired and remains impaired throughout the 6 months of study follow-up both after a single injection of BoNT-A as well as after a booster injection after 3 months^40^. This could most probably be explained by the decreased muscle activity^21,22, 32, 34^ due also to an incomplete recovery of the innervation in the muscle^33,41^. This negatively affects the neuromuscular circuit that regulates the forces acting on the jaw and teeth, the length and speed of contraction of the muscles involved, and the position and speed of the jaw^42^. Consequently, jaw muscle activity (selection function) and bite force (breaking function), determinants of masticatory crushing and grinding, are affected^43^.

As for muscle thickness, muscle activity, and masticatory performance this study indicates that clinicians need to carefully consider not only the beneficial aspects of BoNT-A, but also the possible or potential harms^7^ for treatment of pain and aesthetic purposes.

### Study limitations and strengths

Some limitations of this study should be noted. Firstly, as our study exclusively involved female participants, it is not appropriate to generalize our findings to male individuals, given the different volumetric characteristics of men’s masticatory muscles (which are thicker). Consequently, the dosage of BoNT-A would need to be adjusted for men to achieve positive aesthetic results. Hence, conducting a comparative analysis between genders was not feasible. Moreover, the uniform dosage of BoNT-A administered to all patients in our study, regardless of the severity of MH, inevitably impacted our aesthetic outcomes. In addition, since patients probably are mainly concerned with their appearance during rest, US muscle thickness assessment in rest position was also performed; however, due to technical issues half of the data were lose and were not reported in this study. It is advisable for future studies, to categorize patients based on the severity of MH and adjust the dosage accordingly. Furthermore, comparing our results with other studies proved challenging due to variations in the methodology, including the use of different brands and doses of BoNT-A, as well as differences in the prevalence of more severe MH in other populations (where more pronounced results are expected). In this study we used a total dose of 150 U of abobotulinumtoxinA per patient which is reported to approximates a dose of 60 U of onabotulinumtoxinA. Compared to onabotuliunumtoxinA, which is the most used BoNT-A in US and Europe, abobotulinumtoxinA seems diffuses more easily^26–28^. However, it is also less expensive why it is used in many other parts of the world. To our knowledge, this is the first triple-blinded study using abobotulinumtoxinA to treat MH.

The main strength of our study lies in the longitudinal assessment of the efficacy and adverse effects of both a single and booster injection of BoNT-A, using both subjective and objective evaluations. Additionally, the randomized triple-blinded design employed in our study adds further robustness to our results.

### Conclusion

In conclusion, this study indicates that a single injection of low dose of BoNT-A seems to have a clinically subjective and objective effect on masseteric hypertrophy. However, as a clinician one must consider the possible harms by repeated injections (in shorter intervals), which include impaired masticatory performance, and muscle activity, but also a permanent decrease in muscle thickness. It is still unknown if the changes in neuromuscular function and masseter morphology are permanent and irreversible or have a longer recovery than currently is suggested. Nevertheless, these potential negative effects of BoNT-A must serve as a caution for this treatment, if just for aesthetic purposes.

## Data Availability

The datasets generated during and/or analyzed during the current study are not publicly available but are available from the corresponding author on reasonable request.
